# Integrated analysis reveals common DNA methylation patterns of alcohol-associated cancers: A pan-cancer analysis

**DOI:** 10.3389/fgene.2023.1032683

**Published:** 2023-02-13

**Authors:** Xingyu Liu, Jiarui Chen, Jiali Li, Zihang Zeng, Xueping Jiang, Yanping Gao, Zhengrong Huang, Qiuji Wu, Yan Gong, Conghua Xie

**Affiliations:** ^1^ Department of Radiation and Medical Oncology, Zhongnan Hospital of Wuhan University, Wuhan, China; ^2^ Department of Biological Repositories, Zhongnan Hospital of Wuhan University, Wuhan, China; ^3^ Tumor Precision Diagnosis and Treatment Technology and Translational Medicine, Hubei Engineering Research Center, Zhongnan Hospital of Wuhan University, Wuhan, China; ^4^ Hubei Key Laboratory of Tumor Biological Behaviors, Zhongnan Hospital of Wuhan University, Wuhan, China; ^5^ Hubei Cancer Clinical Study Center, Zhongnan Hospital of Wuhan University, Wuhan, China

**Keywords:** alcohol consumption, alcohol-associated cancer, pan-cancer differentially methylated probe, CpG island, transcription factor motifs

## Abstract

**Background:** The role of alcohol in carcinogenesis has received increasing attention in recent years. Evidence shows its impacts on various aspects, including epigenetics alteration. The DNA methylation patterns underlying alcohol-associated cancers are not fully understood.

**Methods:** We investigated the aberrant DNA methylation patterns in four alcohol-associated cancers based on the Illumina HumanMethylation450 BeadChip. Pearson coefficient correlations were identified between differential methylated CpG probes and annotated genes. Transcriptional factor motifs were enriched and clustered using MEME Suite, and a regulatory network was constructed.

**Results:** In each cancer, differential methylated probes (DMPs) were identified, and 172 hypermethylated and 21 hypomethylated pan-cancer DMPs (PDMPs) were examined further. Annotated genes significantly regulated by PDMPs were investigated and enriched in transcriptional misregulation in cancers. The CpG island *chr19:58220189–58220517* was hypermethylated in all four cancers and silenced in the transcription factor *ZNF154*. Various biological effects were exerted by 33 hypermethylated and seven hypomethylated transcriptional factor motifs grouped into five clusters. Eleven pan-cancer DMPs were identified to be associated with clinical outcomes in the four alcohol-associated cancers, which might provide a potential point of view for clinical outcome prediction.

**Conclusion:** This study provides an integrated insight into DNA methylation patterns in alcohol-associated cancers and reveals the corresponding features, influences, and potential mechanisms.

## 1 Introduction

A strong association has been established between alcohol consumption and carcinogenesis in multiple cancers, including head and neck squamous carcinoma, esophageal squamous carcinoma, hepatocellular carcinoma, breast cancer, and colorectal cancer (Scoccianti et al., 2015). Alcohol consumption is also suspected to associate with pancreatic and lung cancers (Boffetta and Hashibe, 2006). Previous studies have demonstrated a dose-dependent effect of alcohol consumption on survival in head, neck (Lee et al., 2019), and colorectal cancer patients (Cai et al., 2014) and have also demonstrated that alcohol affected tumor development during gene-encoding enzyme alterations for alcohol metabolism, folate metabolism, DNA repair, and oxidative stress (Boffetta and Hashibe, 2006; Seitz and Stickel, 2007). Relationships between alcohol and cancers deserved extensive investigations.

DNA methylation alterations are one of the most frequently identified events in various malignant tumors (Kodach et al., 2010; Lee et al., 2013; Dong et al., 2019) and play important roles in cancer initialization, progression, and recurrence (Ehrlich, 2006; Pellacani et al., 2014; Tahara and Arisawa, 2015; Chen et al., 2016; Das et al., 2019). A group of enzymes known as DNA methyltransferases (DNMTs) are the key regulators that catalyze the methylation process. DNMT1 functions as the maintenance DNMT completing the methylation of the partially methylated DNA, while DNMT3A and DNMT3B catalyze *de novo* methylation. The abnormal expression of DNMTs can silence numerous tumor suppressor genes and affect important biological functions in multiple cancers (Sharma et al., 2010; Fattahi et al., 2018; Zhang et al., 2020). Accumulating evidence has shown that alcohol consumption may cause epigenetic changes, particularly abnormal DNA methylation, which could be important contributory factors to alcohol-induced carcinogenesis (Schernhammer et al., 2010; Boycott et al., 2022; Zhao et al., 2022; Zhou et al., 2022). Alcohol could regulate the expression of DNMTs in mouse models (Mukhopadhyay et al., 2013; Miozzo et al., 2018) and affect DNA methylation-associated pathways *via* reprogramming S-adenosylmethionine (SAMe) metabolism or disturbing the intake of vitamins B6 and B12 (Voigt, 2005; Varela-Rey et al., 2013; Na and Lee, 2017). According to several methylome-wide analyses, a robust association was established between the methylation of specific sites and the clinical outcomes of cancer patients with alcohol consumption (Villanueva et al., 2015; Zhou et al., 2019). Nonetheless, most studies focused on a limited number of CpG sites or genes in a single cancer type, and few analyses rely on the common patterns of alcohol-induced methylome changes. The underlying molecular mechanisms of these aberrant alterations remain elusive, and further explorations are required to uncover their possible effects on tumor biological behaviors.

In this study, we investigated whether a common methylation pattern existed in alcohol-associated neoplasms. The methylome, transcriptome, and clinical information on four alcohol-associated tumors, including esophageal carcinoma (ESCA), head and neck squamous cell carcinoma (HNSCC), liver hepatocellular carcinoma (LIHC), and pancreatic adenocarcinoma (PAAD), was downloaded from The Cancer Genome Atlas (TCGA) portal. The differentially methylated probes (DMPs) were identified between tumors developed due to alcohol consumption and normal tissues. A total of 172 hypermethylated and 21 hypomethylated pan-cancer DMPs (PDMPs) were extracted. The PDMP-annotated genes were investigated and mostly enriched in transcriptional misregulation of cancer pathways. A CpG island *chr19:58220189–58220517* was significantly hypermethylated in all four cancer types and strongly correlated with the downregulation of transcription regulator zinc finger protein 154 (ZNF154), which could serve as a potential epigenetic therapeutic biomarker. Transcription factor (TF) motifs enriched in PDMP regions were clustered into five groups and were significantly associated with multiple tumor hallmarks, on which a TF network was constructed. Furthermore, our studies also identified 11 hypermethylated PDMPs that may be used to predict the overall survival of alcohol-associated cancer patients.

## 2 Methods

### 2.1 Data acquisition

The clinical and transcriptome data on ESCA, HNSCC, LIHC, and PAAD were obtained from TCGA data portal (https://portal.gdc.cancer.gov/). The methylome data were downloaded from the UCSC Xena browser (https://xenabrowser.net/datapages/), which was based on the Illumina HumanMethylation450 BeadChip. GSE123781 is a dataset of oral squamous cell carcinoma patients under alcohol consumption documented in clinical information (Nemeth et al., 2019). The methylation data and clinical data were downloaded from the Gene Expression Omnibus (GEO) database (https://www.ncbi.nlm.nih.gov/geo/).

### 2.2 Data preprocessing

We extracted 129 ESCA, 352 HNSCC, 118 LIHC, and 102 PAAD patients with a clear alcohol consumption history. After samples with no methylation data or gene expression data were excluded, 102 ESCA, 328 HNSCC, 117 LIHC, and 101 PAAD patients were finally applied for further investigation.

Several criteria were used to filter out inappropriate DNA methylation data in this study, including 1) probes having a ‘Not Applicable’ (NA) beta value in more than 50% samples; 2) probes containing no greater than three beads in a minimum of 5% samples; 3) probes with a detection *p*-value ≥ 0.01; 4) multiple-location-hitting probes; 5) probes that are non-CpG; 6) probes associated with single nucleotide polymorphism (SNP) sites; and 7) sex chromosome-specific probes. The k-nearest neighbors (KNNs) method was then applied to estimate the missing value, followed by type II probe normalization using the BMIQ method (Fortin et al., 2017). Using the *ChAMP* package, the aforementioned steps were performed to preprocess raw methylation data (Morris et al., 2014).

### 2.3 DMP and PDMP definitions

The Limma method in the ChAMP package was applied to identify DMPs in each cancer. The threshold of a significant probe is the fold change (FC) of a beta value > 1.2 and *p*-value < 0.05. A DMP with a higher methylation level (beta value) in the tumor sample was defined as hypermethylated DMP, while a DMP with a lower methylation level was defined as hypomethylated DMP. A probe is defined as PDMP if it can be identified as DMP in all four cancer types and has the same methylation status (hypermethylated or hypomethylated).

### 2.4 Regional methylation status assessment

Using probe beta values for one cancer type in a specific region, we investigated the methylation status of various chromosomes and functional CpG regions. The formula is as follows:

$$MS=median\left(\sum M_{i,T}^{p,r}\right)-median\left(\sum M_{j,N}^{p,r}\right).$$

For a given cancer type, the median beta value M of probe *p* in region *r* of all tumor samples *T* or normal samples *N* was calculated. A hypermethylated region was defined as having a *p*-value < 0.05 and MS > 0, while a hypomethylated region was defined as having a *p*-value < 0.05 and MS < 0.

### 2.5 Correlation between PDMPs and gene expression

We calculated the Pearson coefficient correlations (PCCs) between PDMPs and their annotated genes. A significant correlation was defined as having |PCC| > 0.2 and *p*-value < 0.05. Because the positions of CpG probes in different regions may have a different effect on genes (Yang et al., 2015), probe regions were divided into two groups: a) the promotor region, consisting of *TSS200*, *TSS1500*, *5′UTR*, and *1stExon*, and b) the body region, consisting of *Body*, *3′UTR*, and *IGR*. Upstream and downstream genes that may potentially be regulated by enhancers located on specific probes were investigated using the ELMER package (Silva et al., 2019). A significant correlation was defined as having |PCC| > 0.1 and *p*-value < 0.05.

### 2.6 Motif enrichment analysis

As a measurement of TF-binding motifs enriched in PDMP regions, we generated sequences of 500 bp between 250 bp upstream and 250 bp downstream of each probe and divided them into hypermethylated and hypomethylated groups, followed by motif enrichment analysis using the AME function on the MEME website (http://meme-suite.org/tools/ame) (Buske et al., 2010; Bailey et al., 2009; Kulakovskiy et al., 2018). The TF-enriched motif was obtained with a *E-*value < 10^−5^, which was subsequently clustered using STAMP (http://www.benoslab.pitt.edu/stamp/index.php) (Mahony and Benos, 2007). *clusterProfiler* was used to explore pathways enriched in the Kyoto Encyclopedia of Genes and Genomes (KEGG) (Yu et al., 2012).

### 2.7 Construction of the TF-regulated network

The TRRUST database (https://www.grnpedia.org/trrust/) is a practical website for TF-gene interaction prediction, collecting 8,444 TF-target regulatory relationships derived from 11,237 experiment-based articles (Han et al., 2018), which was used to screen and filter high-reliability TF-gene pairs. Subsequently, 40 TFs and their target genes were selected to construct a TF-regulated network in each cancer based on the adjacent matrix created by the weighted method (Langfelder and Horvath, 2008). Each cancer regulatory network’s top 10% weighted edges, which represent TF-gene interaction intensity, were combined into one pan-cancer regulatory network (Yu, 2020). Genes were clustered based on the greedy algorithm (Girvan and Newman, 2002).

### 2.8 Survival analysis

To evaluate the prognostic ability of PDMPs for each cancer type, a univariate Cox regression model was applied. Probes with *p*-value < 0.1 in at least three cancer types were extracted and considered prognosis-related PDMPs, followed by multivariate Cox analysis. For each cancer, we evaluated the methylation prognosis index (MPI) of each sample and divided them into high- and low-risk groups by the following formula:

$${MPI}_{i}=\overset{n}{\underset{p}{\sum }}\beta_{p,i}I_{p}.$$

For a given sample *i*, the beta value of prognosis-related PDMP *p* to the index coefficient *p* was summed. To evaluate the survival difference between two groups of patients, Kaplan–Meier survival analysis was performed. AUC was calculated using the *timeROC* package of R software to validate the predictive ability of the MPI model (Blanche et al., 2013).

### 2.9 Statistical analysis

The statistical significance of continuous variables was estimated using the Wilcoxon rank-sum test. Univariate and multivariate Cox regression analyses were performed to construct the MPI model using the *survival* package (Holleczek and Brenner, 2013). All the aforementioned statistics were analyzed by R software (version 4.0.2).

## 3 Results

### 3.1 Characteristics of alcohol-associated DNA methylation patterns

**FIGURE 1:**
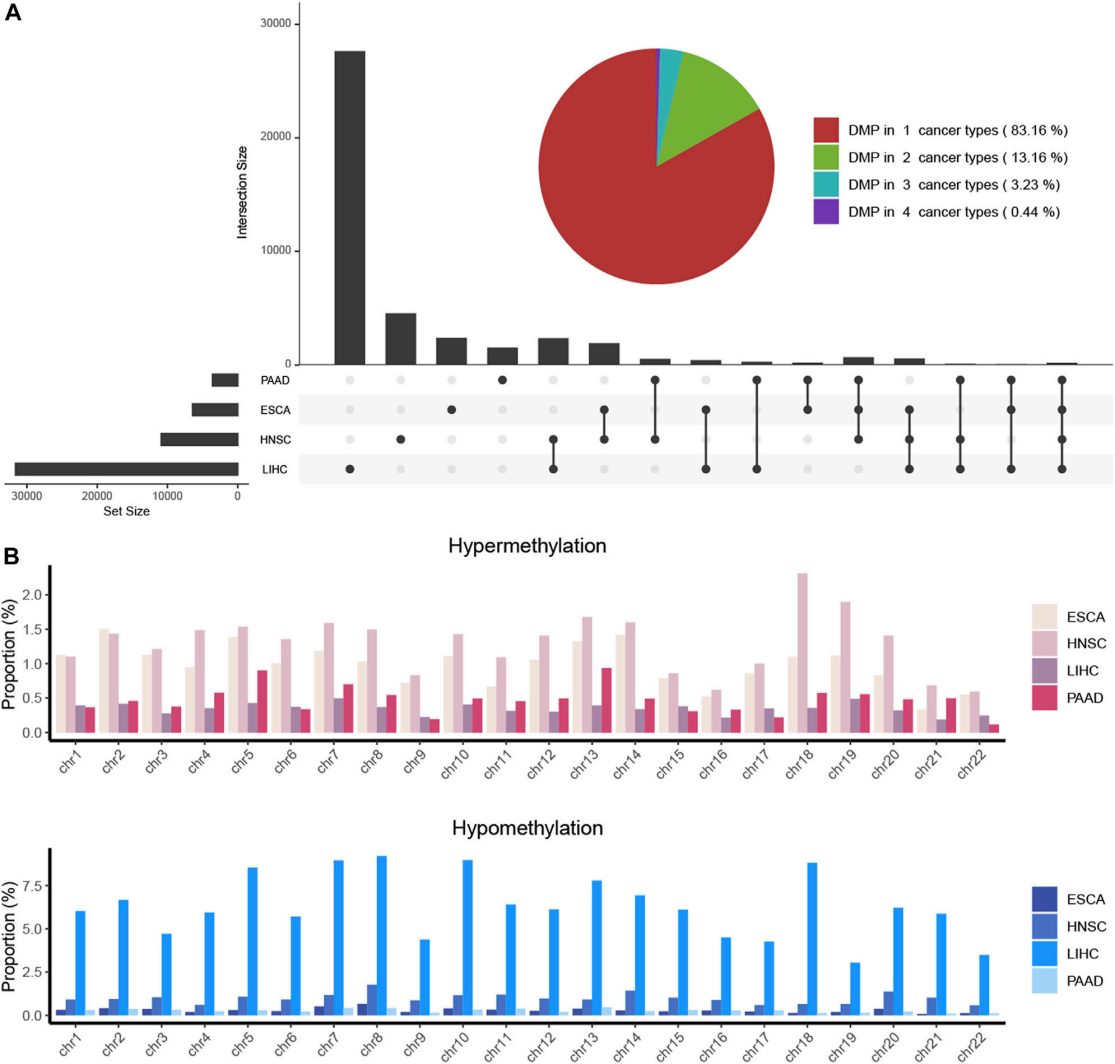
DMPs were investigated in four alcohol-associated cancers. **(A)** Combined plot showed the number of DMPs in each cancer type and that of multiple cancer types. The number of the same DMPs between different cancer types was calculated and shown as the intersection size. **(B)**. Proportion of hypermethylated and hypomethylated DMPs located in chromosomes of each cancer type. The proportion was determined as the ratio of the number of identified DMPs to the total number of CpG probes on each chromosome.

**FIGURE 2:**
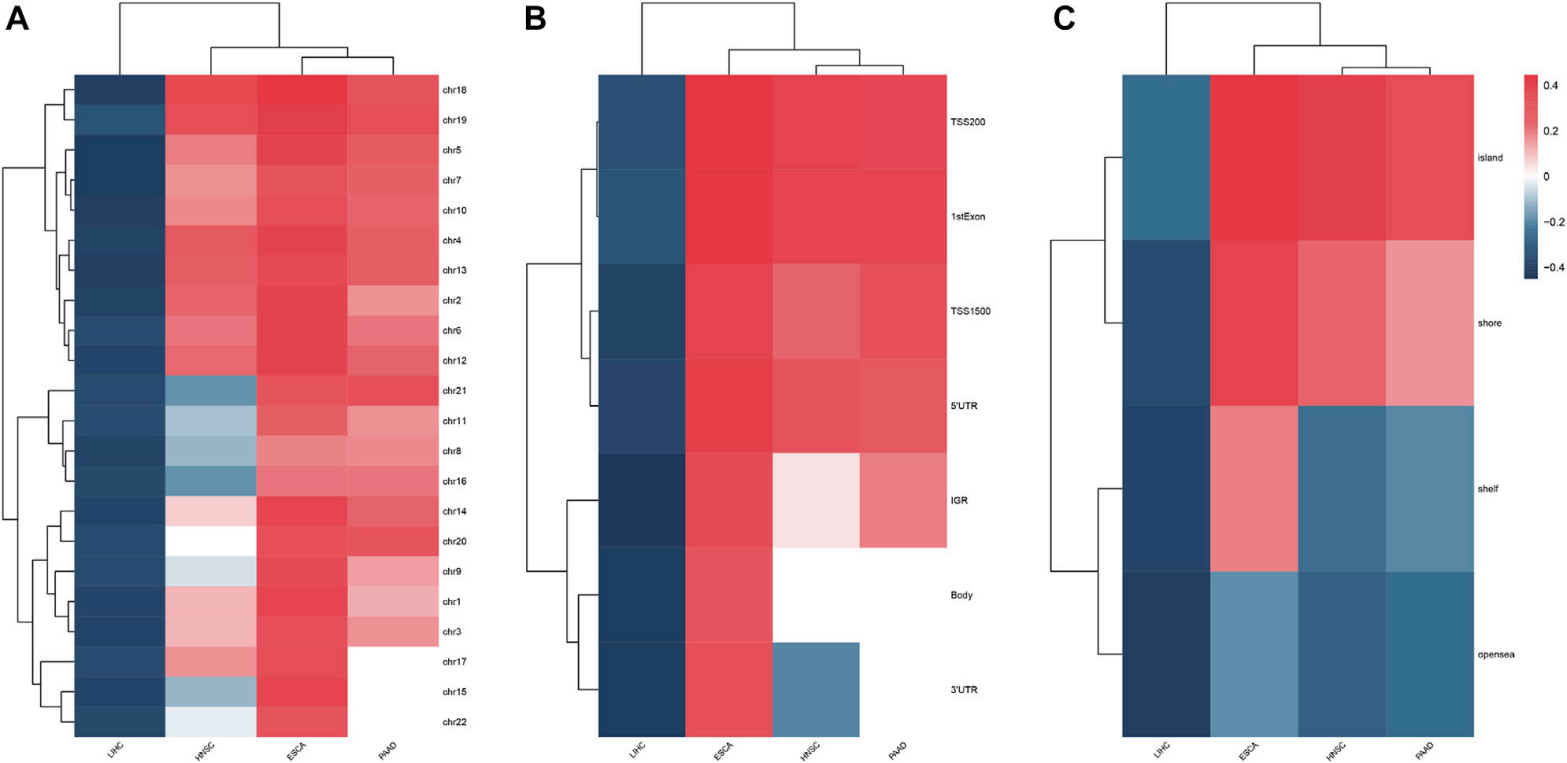
Heatmap showed the methylation status of chromosomes **(A)**, feature regions **(B)**, and CpG regions **(C)** in four alcohol-associated cancers.

The clinical information on each alcohol-associated aforementioned cancer was downloaded from TCGA database. ESCA, HNSCC, LIHC, and PAAD were included in further studies, and other alcohol-related cancer types, such as lung and colon cancer, were excluded due to the absence of a documented history of alcohol consumption. We identified a set of DMPs in four cancers, with 6,436 in ESCA, 10,891 in HNSCC, 31,620 in LIHC, and 3,592 in PAAD patients (Figure 1A), and the proportion of hypermethylated or hypomethylated DMPs distributed on each normal chromosome to all probes in that region was measured (Figure 1B). Given the number of DMPs in each cancer, DNA methylation alterations in pancreatic cancer might be affected slightly by alcohol consumption, whereas liver cancer was more affected. The methylation status of chromosomes in ESCA and PAAD separately was more hypermethylated or hypomethylated and that of HNSCC and LIHC was in an intermediate status (Figure 2A). Most DMPs tended to be located in non-promotor regions (Supplementary Figure S1A, S1B) and an approximately equal percentage of the CpG island and open-sea regions (Supplementary Figure S1C, S1D). Functional regions were categorized based on the methylation status into two groups, namely, the promotor (*TSS200*, *TSS1500*, *5′UTR*, and *1stExon*) and body region (*Body*, *3′UTR*, and *IGR*) or CGI (*CpG island*), and non-CGI region (*CpG shore*, *shelf*, and *opensea*) (Figures 2B,C), each of which was annotated with biological functions.

**FIGURE 3:**
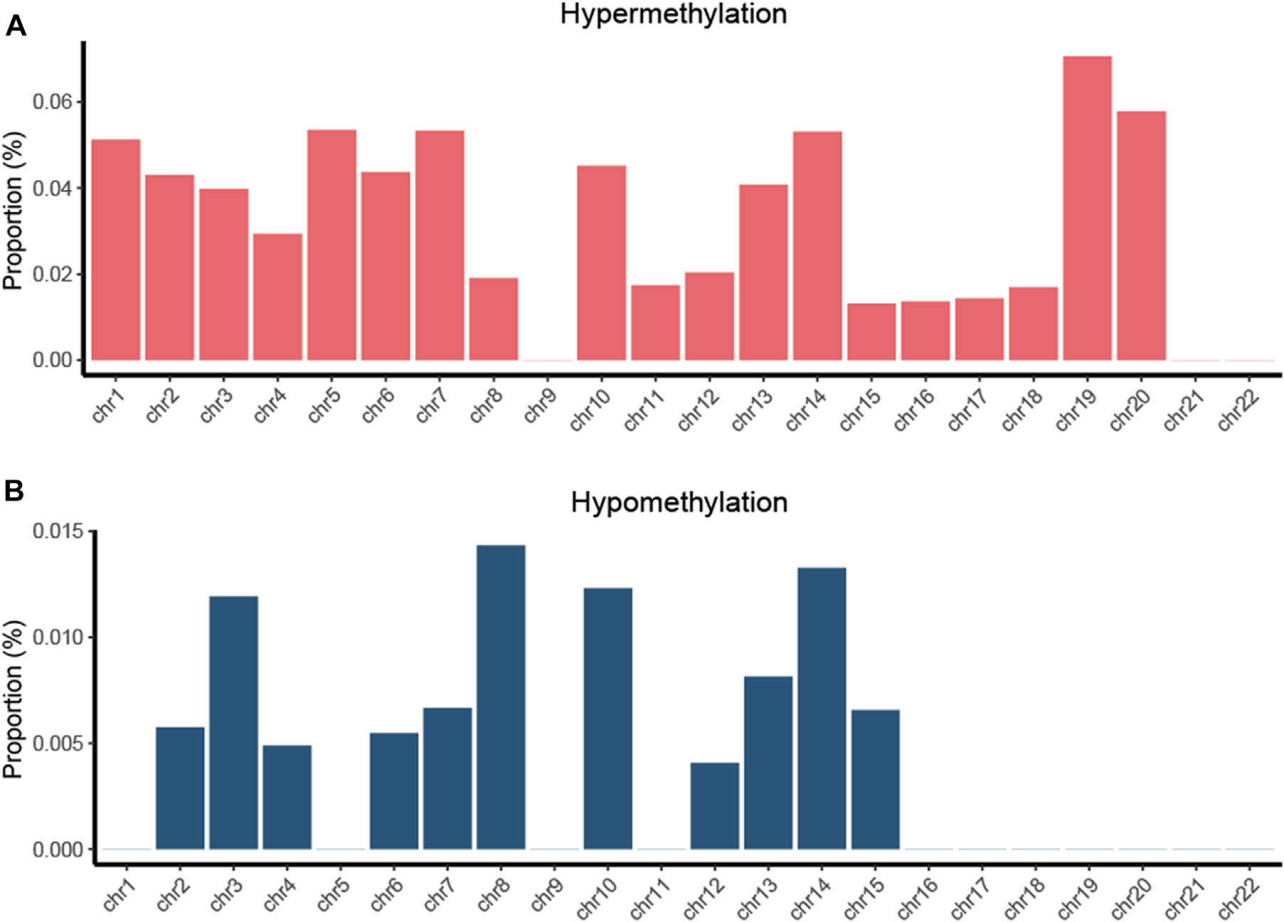
Proportion of hypermethylated **(A)** and hypomethylated **(B)** PDMPs located in chromosomes. The proportion was determined as the ratio of the number of identified PDMPs to the total number of CpG probes on each chromosome.

We computed DMPs with the same methylation status in all four types of cancers and obtained 172 hypermethylated and 21 hypomethylated PDMPs (Supplementary Figure S2A, S2B). The highest proportion of hypermethylated PDMPs was found on chromosome 19, while the highest proportion of hypomethylated PDMPs was found on chromosome 8. (Figure 3). Interestingly, there was no PDMP located in chromosomes 9, 21, and 22. One of the possible explanations could be that DMPs in these chromosomes exhibit biological behaviors that are specific to cancer. Compared with distribution of the functional region of DMPs, PDMPs located in promotor or body regions showed an almost equal percentage distribution (46.7% and 53.3%) (Supplementary Figure S3A, S3B). In addition, 72% of PDMPs were located in the CGI region (Supplementary Figure S3C, S3D). We also validated the PDMPs in GSE123781, and 182 of 193 probes were found significantly differentially methylated (Supplementary Table S1). Taken together, alcohol-associated aberrant pan-cancer methylation changes were more likely to exert biological effects in promotor and CGI regions.

Aging was suggested to be associated with the increase of methylation at a global level (Maegawa et al., 2017). To identify whether aging contributed to the alcohol-associated methylation alterations in our study, we investigated the DMPs between the young group (<65 years old) and the old group (≥65 years old). We found that there was only a total of 26 CpG probes with an adjusted *p*-value < 0.05, with 21 in ESCA, 1 in HNSCC, 4 in LIHC, and none in PAAD patients (Supplementary Table S2). Interestingly, none of these probes reached the DMP-defined threshold of fold change > 1.2. These results suggest that aging has a very slight impact on our findings.

### 3.2 PDMPs regulated the expression of annotated genes

**FIGURE 4:**
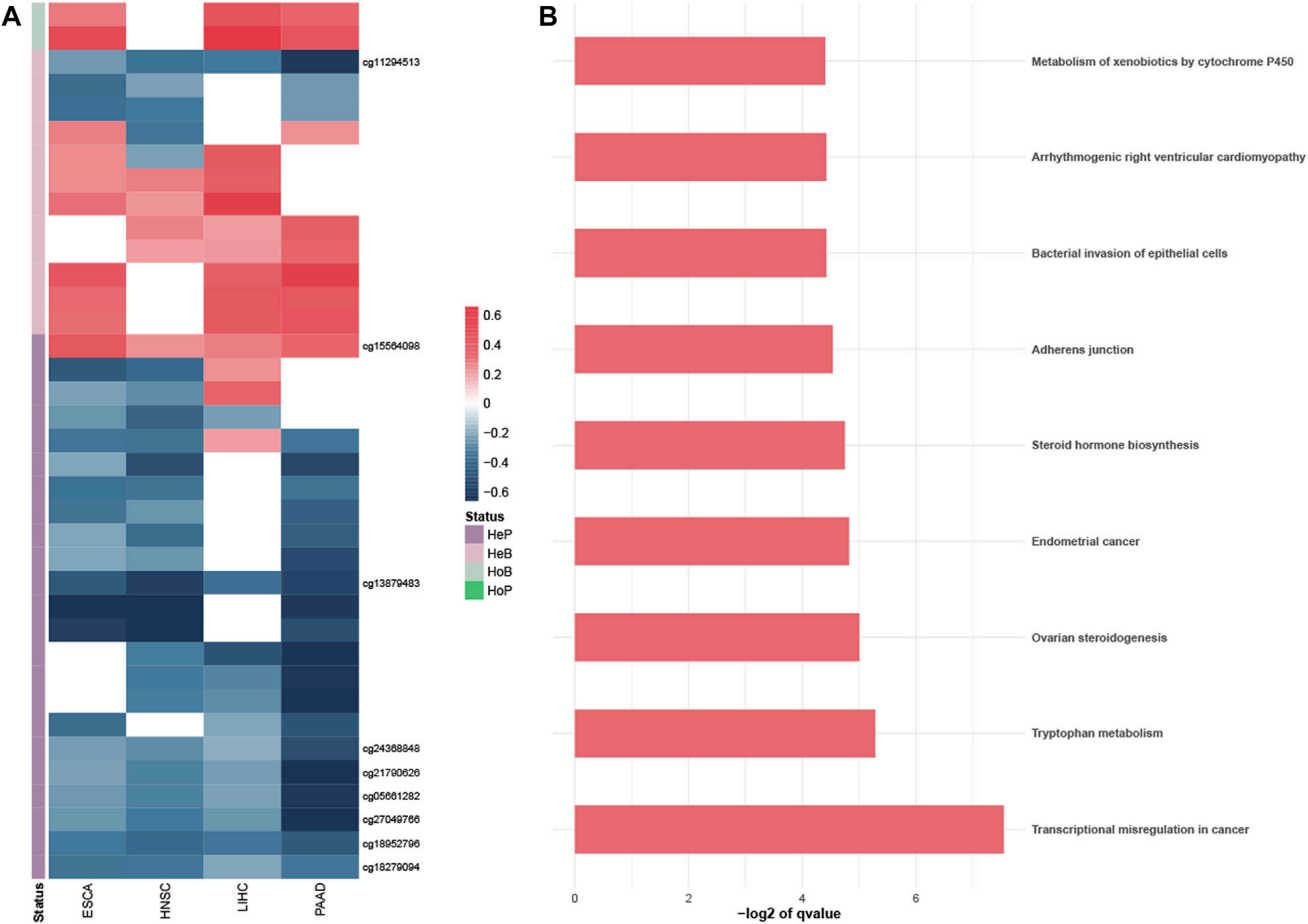
Exploration of the PDMP-annotated genes. **(A)** Heatmap revealed the Pearson coefficient correlations between PDMPs and annotated genes. PDMPs were defined into four groups: HeP, hypermethylated probe in the promotor region; HeB, hypermethylated probe in the body region; HoP, hypomethylated probe in the promotor region; HoB, hypomethylated probe in the body region. **(B)**. KEGG enrichment analysis of 33 PDMP-annotated genes.

To better understand the potential biological effect of these PDMPs, PCCs were used to evaluate the correlation between PDMPs and their annotated downstream genes. Since probes with different relative locations may have a different regulatory effect on expression, 193 PDMPs were defined into four groups: HeP = hypermethylated probe in the promotor region, HeB = hypermethylated probe in the body region, HoP = hypomethylated probe in the promotor region, and HoB = hypomethylated probe in the body region. Thirty-seven PDMP–gene pairs were identified in at least three cancer types, and nine PDMP–gene pairs were found in all cancers (Figure 4A; Supplementary Table S3). These 37 PDMP-annotated genes were enriched in several cancer hallmarks, among which *transcriptional misregulation in cancer* (q-value = 5.35e^−3^) stood out as the most significant one (Figure 4B). There was a surprising outcome that four of nine aforementioned PDMPs (*cg01268824*, *cg11294513*, *cg21790626*, and *cg27049766*) belonged to the same CpG island *chr19:58220189-58220517* and correlated with the same gene *ZNF154*. The role of this CpG island required further exploration.

### 3.3 Hypermethylated CpG island *chr19:58220189-58220517* silenced downstream gene expression

**FIGURE 5:**
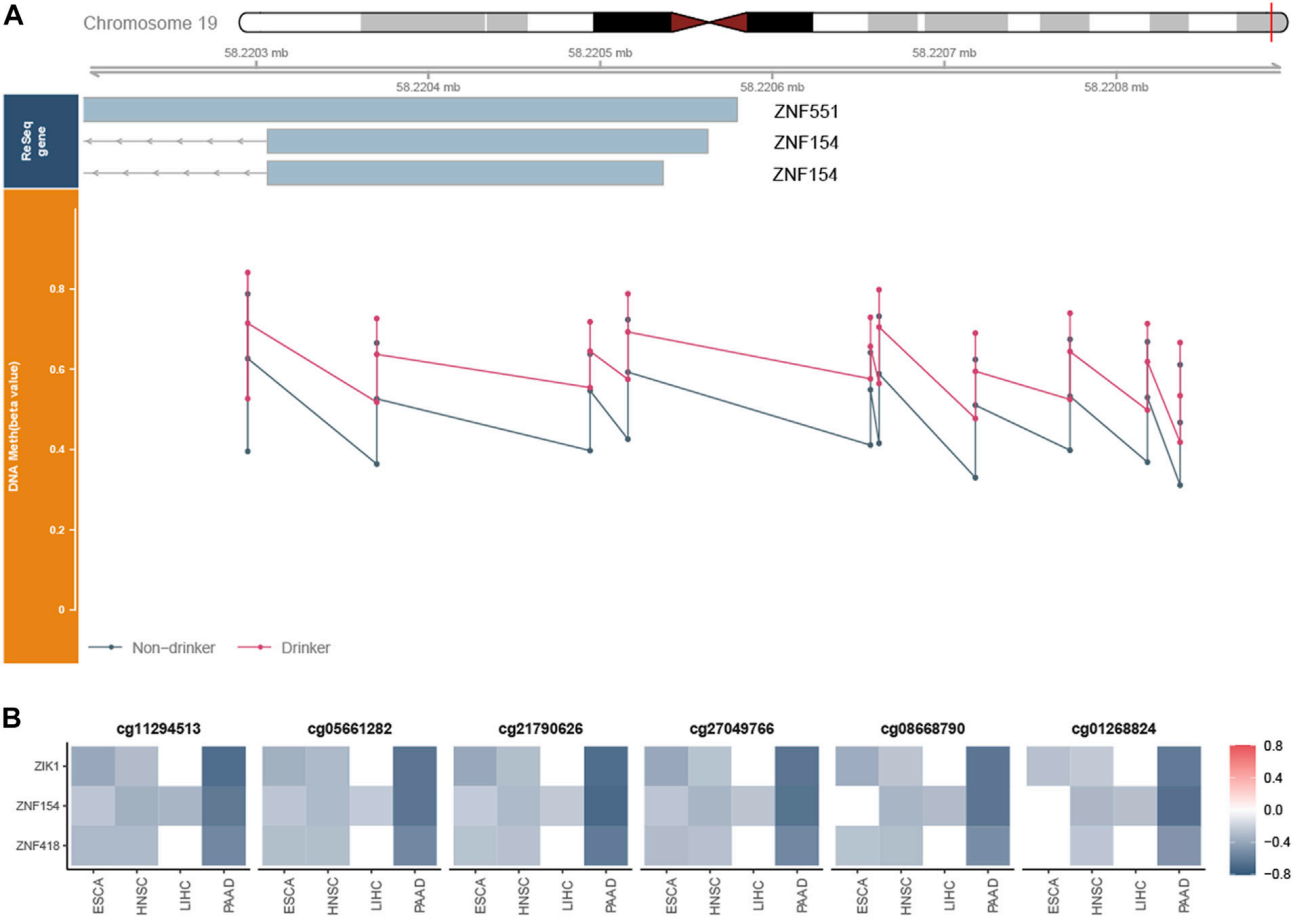
Exploration of the CpG island *chr19:58220189–58220517*. **(A)** Whole view of CpG probes in the CpG island *chr19:58220189–58220517* demonstrated its hypermethylation status. The bottom dot plots from left to right represent the beta values of cg1129451, cg0566128, cg21790626, cg27049766, cg03234186, cg08668790, cg12506930, cg26465391, cg01268824, and cg27324426, respectively. *cg03142586* was excluded from further analysis since its beta value was NA in most samples. **(B)** Heatmap revealed the correlation between the beta value of six PDMPs and the expression of *ZNF154*, *ZIK1*, and *ZNF418* in four cancer types.

We checked all probes in *chr19:58220189–58220517* and found a total set of 11 probes, among which six probes were PDMPs (*cg11294513*, *cg05661282*, *cg21790626*, *cg27049766*, *cg08668790*, and *cg01268824*). *cg03142586* was excluded from further analysis since its beta value was NA in most samples. Probes in this region were remarkably hypermethylated (Figure 5A; Supplementary Figure S4), which were validated in GSE123781 (Supplementary Figure S5). It has been shown that exposure to alcohol increases methylation levels at certain loci in normal tissues (Lee et al., 2011). To obtain an overall view of the methylation status of the region, we investigated the beta values of samples exposed or not exposed to alcohol. Significant differences were observed between alcohol-associated tumor samples and non-alcohol-associated tumor samples (Supplementary Figure S6). A strong negative correlation was found between the beta values of these PDMPs and the expression of *ZNF154* (Figure 5B), suggesting a potential silence effect. Recently, the methylome and transcriptome-based algorithm has been developed to identify cancer-specific enhancers (Yao et al., 2015). The CpG probes located on these enhancers possess the potential ability to regulate the expression of upstream and downstream genes. We then applied this method and explored the relationship between these PDMPs and the closest 10 upstream and downstream genes (Supplementary Figure S7). Together with *ZNF154*, zinc finger protein interacting with K protein 1 (*ZIK1*, the seventh closest upstream gene) and *ZNF418* (the ninth closest downstream gene) was also found to have a negative correlation with six PDMPs (Figure 5B), suggesting the hypermethylation status of the CpG island *chr19:58220189–58220517* could potentially mediate transcription activity of these three genes. All three genes were associated with DNA-binding activity, suggesting that the disorder of DNA recognition might be involved in alcohol-associated tumor behaviors.

### 3.4 Different pathways are correlated with TF motifs in PDMP regions

**FIGURE 6:**
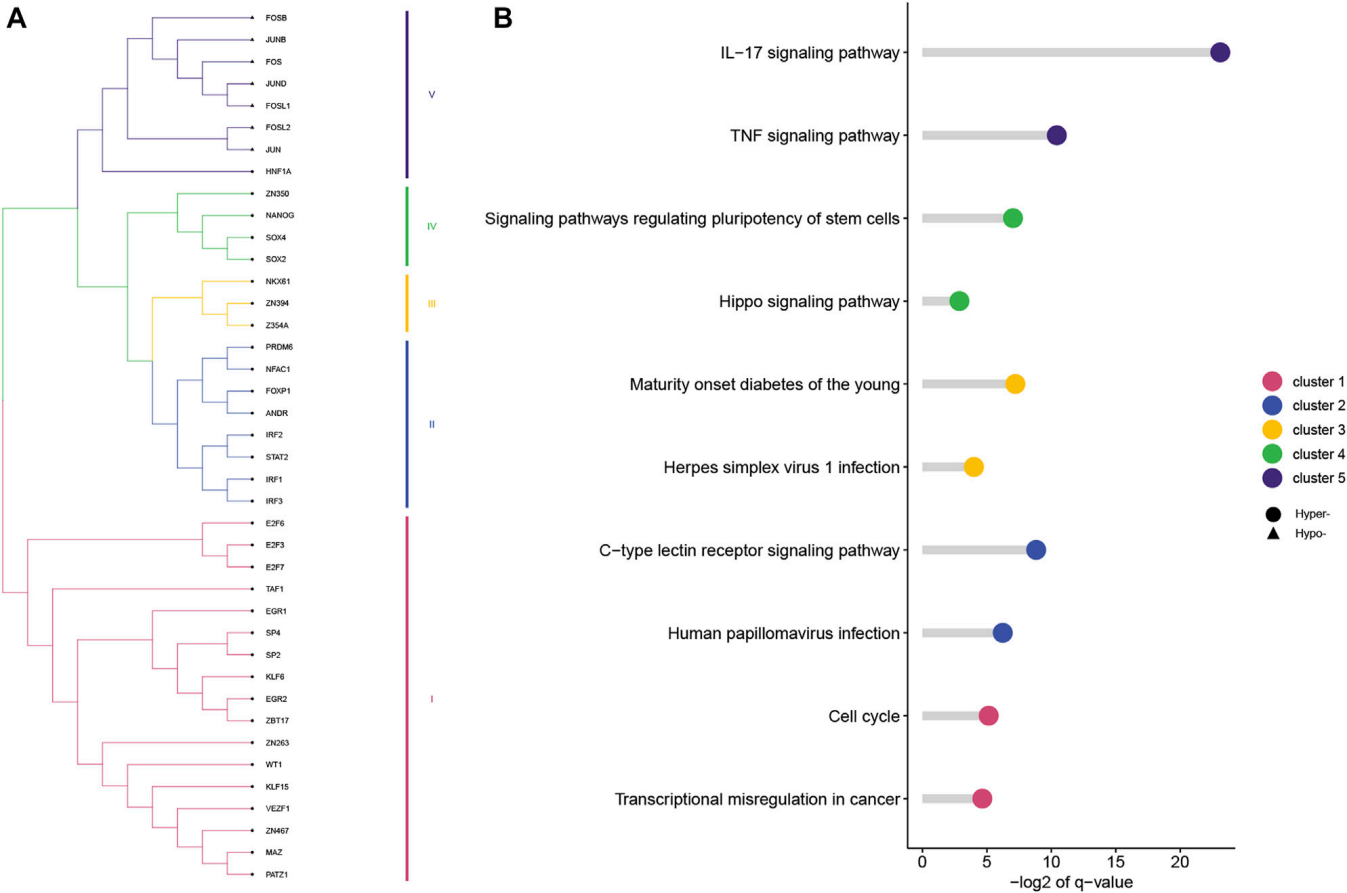
TF motifs enriched in hypermethylated and hypomethylated PDMPs. **(A)** TF motifs were divided into five clusters based on the sequence similarity. **(B)** KEGG enrichment analysis of five clusters. The top two enriched pathways of each cluster were visualized.

**FIGURE 7:**
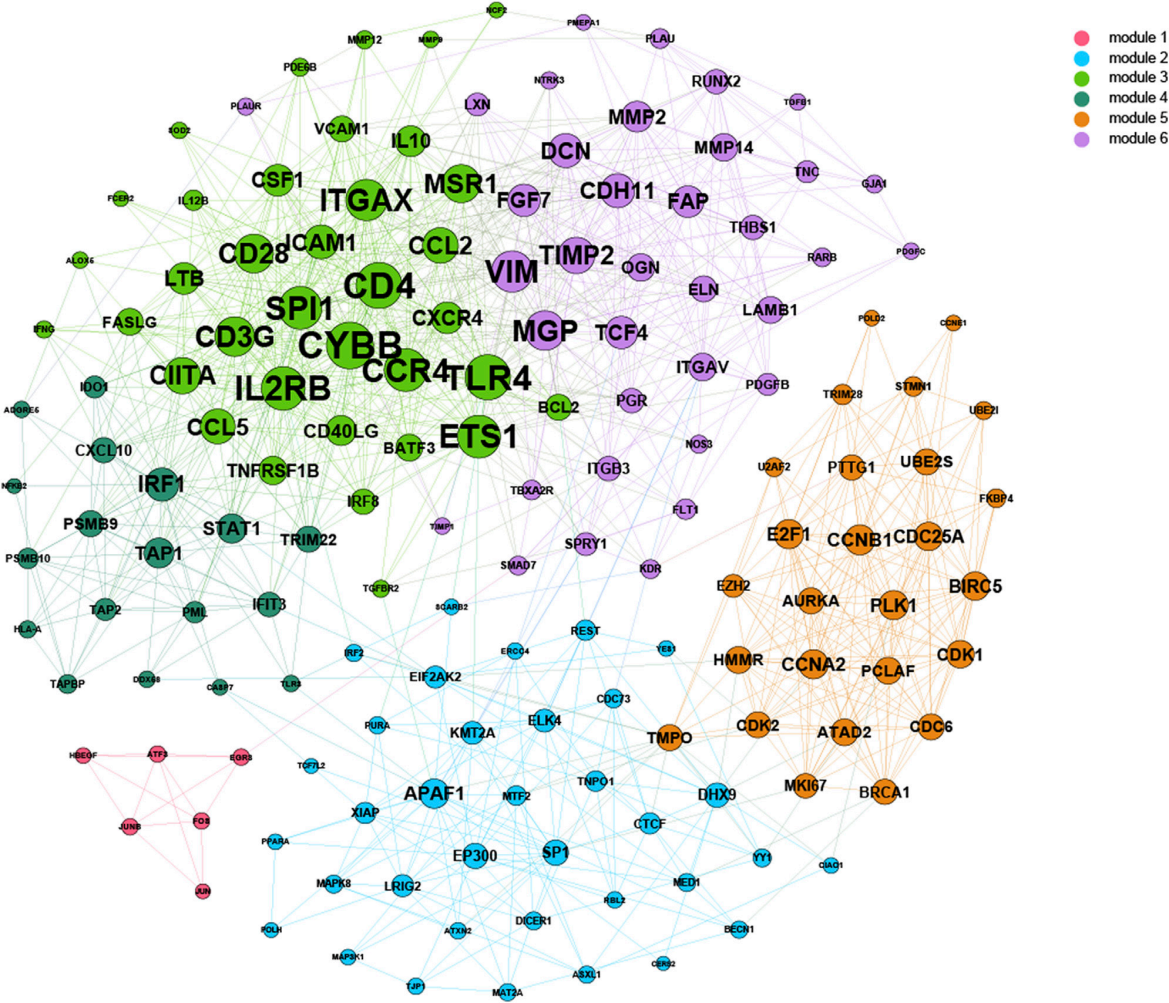
Construction of the combined TF-gene regulatory network. For each cancer, a regulatory network was constructed from the adjacent matrix. The top 10% of TF-gene interactions were considered significant and combined into one network.

Based on 500-bp sequences generated from PDMPs, possible TF-binding motifs were scanned, enriched, and divided into hypermethylated and hypomethylated groups. Thirty-three and seven motifs located in hypermethylated and hypomethylated regions, respectively, were identified, followed by the grouping of these motifs into five clusters with STAMP. (Figure 6A; Supplementary Table S4). POZ/BTB and AT-hook-containing zinc finger protein 1 (*PATZ1*) showed the most significant enrichment in the hypermethylated group (*E*-value = 3.36e^−17^), and among hypomethylated groups, *JUN* was identified as the most significant motif (*E*-value = 6.47e^−17^). All seven hypomethylated motifs shared a close relatedness and were clustered into one group, suggesting a similar regulatory mechanism among them. The motif clusters exhibited distinct biological pathways, and several pathways appeared to be involved in the development and differentiation of cancers. (Figure 6B). Cluster I was significantly enriched in the *cell cycle* (*q*-value = 2.80e^−2^), and Cluster IV was associated with *signaling pathways regulating the pluripotency of stem cells* (*q* = 7.00e^−3^). Additionally, Cluster IV was associated with the *IL-17 signaling pathway* (*q*-value = 1.10e^−7^) and *TNF signaling pathway* (*q*-value = 7.28e^−4^), which is possibly involved in modulation of the immune response. According to the TRRUST database, we further explored the target genes associated with the aforementioned TF motifs and constructed a transcriptional regulatory network, which was recognized as six distinct modules (Figure 7).

### 3.5 The prognostic potential of PDMPs

**FIGURE 8:**
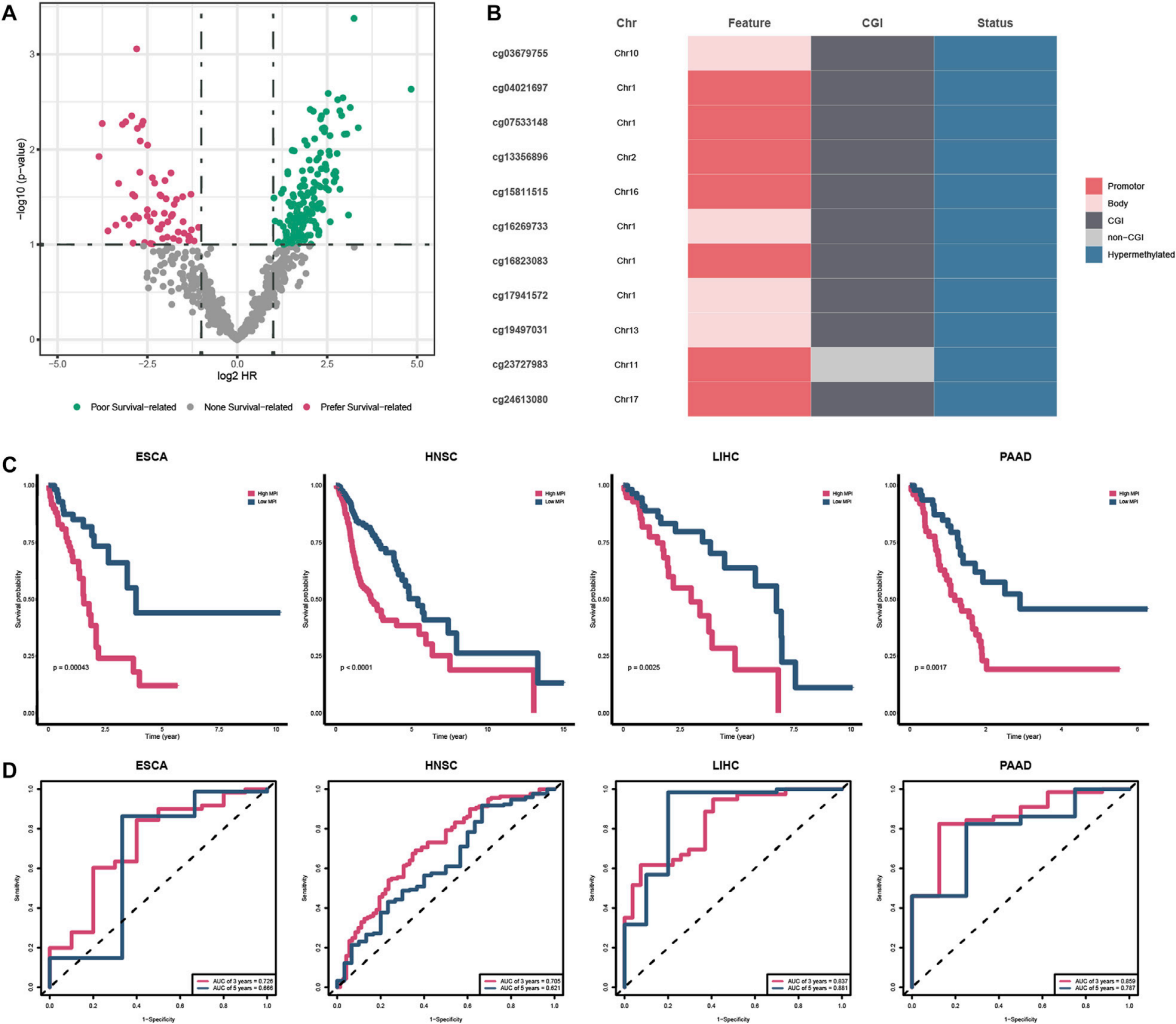
Survival analysis of PDMPs. **(A)** Volcano plot showing the survival-related PDMPs in each cancer. **(B)**. Characteristics of 11 survival-related PDMPs. **(C)** Kaplan–Meier analysis of high- and low-MPI group patients in each cancer type. **(D)**. ROC curve validating the predictive ability of MPIs.

To evaluate the prognostic value of specific PDMPs, we performed univariate Cox regression analyses in each cancer separately (Figure 8A). We screened 11 PDMPs as they were associated with prognosis in at least three types of cancer, and these PDMPs were all hypermethylated (Figure 8B; Supplementary Table S5). Seven out of 11 PDMPs (63.6%) were found in promoter regions, while 10 out of 11 (90.1%) were found in CGI regions, which confirmed the aforementioned findings that alcohol-associated PDMPs were more likely to exert biological effects on these regions. Patients were classified into low- and high-MPI groups in each cancer based on the MPIs. High-MPI groups were significantly associated with a worse outcome than low-MPI groups by Kaplan–Meier survival analysis (Figure 8C). The ROC curves were obtained, and the AUC of 3-year OS (range from 0.705 to 0.859) and 5-year OS (range from 0.621 to 0.881) was calculated separately, revealing the reliability of MPIs (Figure 8D).

## 4 Discussion

The purpose of this study was to investigate PDMPs in four cancers that were associated with alcohol consumption and the distribution of PDMPs within these cancers. Promotor and CGI regions were the two main regions that PDMPs were more likely to enrich. Thirty-seven PDMPs were found to show a close regulatory relationship with annotated genes and correlate with transcriptional misregulation. Furthermore, we observed a hypermethylated CpG island *chr19:58220189–58220517*, showing a strong correlation with transcription regulatory genes. Thirty-three hypermethylated and seven hypomethylated TF motifs were clustered into five groups and exerted various biological effects. Finally, 11 PDMPs were reported to be associated with overall survival, providing valuable prediction of clinical outcomes.

Many DMPs were found in each of the four alcohol-associated cancers, and only a small percentage of DMPs were shared, suggesting the presence of cancer-specific methylation patterns. A study by Fan et al., for instance, revealed that preferred genomic DNA sequence patterns were observed between different DNMTs in hepatocellular carcinoma cells, which might promote to the formation of a hepatocellular carcinoma-specific methylation landscape (Fan et al., 2016). Cancer-specific methylation patterns have not been explored here due to the limited scope of our studies on common patterns of altered DNA methylation, which requires further study.

The genome methylation landscape is wholly poor of CpG and locally rich (in CpG islands). CGIs could be found in more than half of the genes in the vertebrate genome and commonly maintain a low methylated level (Jones, 2012). We reported a hypermethylated CpG island *chr19:58220189–58220517* in all four alcohol-associated cancers, and this finding is consistent with that of Ruike Y et al., who also found that CGI regions tend to be hypermethylated in cancers (Ruike et al., 2010). The expression of *ZNF154* was significantly downregulated by the CpG island *chr19:58220189–58220517*, which has been partially demonstrated by previous studies. Based on a computational algorithm, we also found that the transcription activity of *ZIK1* and *ZNF418* might be regulated by hypermethylation of the CpG island *chr19:58220189–58220517.* According to a blood-based diagnostic model, hypermethylation of the *ZNF154* CpG island was identified as a relevant biomarker for detecting circulating solid tumor DNA (Margolin et al., 2016). Epigenetic silencing of *ZNF154* was associated with multiple cancers and could serve as a biomarker predicting the recurrence of alcohol-associated pancreatic cancer (Yamada et al., 2016; Mishra et al., 2019; Wiesmueller et al., 2019), as well as non-alcohol-associated prostate cancer (Nahar et al., 2018) and bladder cancer (Reinert et al., 2012). Hu et al. showed that *ZNF154* might serve as a tumor suppressor, and overexpression of *ZNF154* significantly inhibited cell migration and invasion *in vivo,* as well as lung nodule formation *in vitro, via* suppressing Wnt/β-catenin signaling pathway activation (Hu et al., 2017). *ZIK1* was identified as a transcriptional repressor blocking a gene promotor bearing Gal4-binding elements *via* interacting with heterogeneous nuclear ribonucleoprotein particle K protein (Denisenko et al., 1996). Silenced *ZIK1* was observed in noncancerous esophageal mucosae (Oka et al., 2009), suggesting low expression of *ZIK1* occurred at the early stages of cancer. Only a few studies documented that *ZNF418* negatively regulated transcription and the MAPK signaling pathway (Li et al., 2008) and that lower expression of *ZNF418* was associated with poorer prognosis in gastric cancer (Hui et al., 2018). As our scope was limited to methylation alterations, the role of associated genes was not identified, which required further investigation.

Thirty-three and seven TF motifs located in hypermethylated and hypomethylated PDMP regions, respectively, were identified as transcription regulators in alcohol-associated cancers, of which *PATZ1* and *JUN* were most notable. *PATZ1*, containing an A–T hook DNA-binding motif, binds to other DNA-binding structures to participate in chromatin modeling and transcription regulation. *PATZ1* is involved in the inhibition of the mesenchymal-to-epithelial transition *via* disturbing the combination between p53 gene and its response elements (Chiappetta et al., 2015; Keskin et al., 2015) and could be an independent prognostic factor in multiple cancers (Guadagno et al., 2017; Zhao et al., 2018; Passariello et al., 2019). *Jun* was involved in a number of biological processes that were triggered by oxidants and toxic stimuli when combined with other members of the Fos family (Angel and Karin, 1991; Shaulian and Karin, 2002). An *in vitro* experiment showed that alcohol promotes the direct recruitment of *c-Jun* to TATA-binding protein, *Brf1*, and tRNA gene promotors, inducing RNA polymerase III-dependent transcription, thus contributing to liver tumor development (Zhong et al., 2011). We established a TF-gene regulatory network in our article that might assist in understanding the regulatory functions and mechanisms of alcohol and carcinogenesis.

Aging may also contribute to methylation changes. Several studies indicated that aging was closely linked to global hypermethylation. In normal breast tissues, age acceleration was observed (Hofstatter et al., 2018).The methylation level of highly variable sites increased from a mean of 3% in the newborn to 20% in the old, and these age-related hypermethylation cases were found to be enriched in the CGI region (Maegawa et al., 2017). A large-scale methylation study revealed that cancer risks and mortality increased with aging epigenetic changes, ranging from 4% to 9% and 2% to 6%, respectively, with 5 years of age acceleration (Dugue et al., 2018). According to Zheng et al., most epigenetic drifts were nonfunctional, while some might randomly affect TF expression or the binding affinity, resulting in an abnormality in tissue homeostasis (Zheng et al., 2016). In light of the complex function and mechanism underpinning aging and tumor epigenetics, the effect of aging on PDMPs identified in this article has been difficult to estimate and remove.

This study only examined a small part of methylation sites. Although the Illumina HumanMethylation450 array provided coverage of 98.9% UCSC RefGenes, the coverage rate of total genome CpG sites was only 2% (Bibikova et al., 2011; Plongthongkum et al., 2014), leading to incomplete exploration of alcohol-associated genome-aberrant CpG patterns. Non-coding RNAs also play significant roles in cancer initiation, progression, and metastasis (Gupta et al., 2010; Li et al., 2015; Ferreira and Esteller, 2018). The function and correlation of CpG sites annotated in these regions were not explored. Due to a lack of alcohol consumption documents in TCGA database, other alcohol-associated cancers such as colorectal and lung cancers were not included in this study.

## 5 Conclusion

Based on multi-omics data on pan-cancer, this study explored the global DNA methylation alterations of ESCA, HNSCC, LIHC, and PAAD patients with alcohol consumption documents from TCGA database. A total of 193 PDMPs were identified, and the preference patterns of alcohol-associated DNA methylation changes were located in promotor and CGI regions. PDMP-annotated genes were enriched in multiple pathways, especially transcriptional misregulation in cancer, demonstrating that alcohol might contribute to transcriptional disorder by inducing the methylation status of transcription regulators, thus leading to tumor development. A hypermethylated CpG island *chr19:58220189–58220517* was identified, and it regulated the transcription activity of downstream genes, serving as a potential therapeutic biomarker. Five sets of enriched TF motifs were involved in numerous cancer hallmarks including tumorigenesis and immunoregulation, and a TF-gene regulatory network was constructed for a better understanding of potential regulation mechanisms. Additionally, 11 PDMPs were reported to be associated with the overall survival of patients. Estimated MPIs are reliable and provide a potential point of view for clinical outcome prediction.

## Data Availability

Publicly available datasets were analyzed in this study. These data can be found at: TCGA (https://cancergenome.nih.gov) and GEO (https://www.ncbi.nlm.nih.gov/geo/) repositories.

## Abbreviations

DMPs, differential methylated probes; PDMPs, pan-cancer differential methylated probes; ESCA, esophageal carcinoma; HNSCC, head and neck squamous cell carcinoma; LIHC, liver hepatocellular carcinoma; PAAD, pancreatic adenocarcinoma; ZNF154, zinc finger protein 154; TF, transcription factor; FC, fold change; PCCs, Pearson coefficient correlations; KEGG, Kyoto Encyclopedia of Genes and Genomes; MPI, methylation prognosis index; AUC, area under the curve; HeP, hypermethylated promotor; HeB, hypermethylated body; HoP, hypomethylated promotor; HoB, hypomethylated body; PATZ1, POZ/BTB and AT-hook-containing zinc finger protein 1.
