# Supplementary material for: Integrated analysis reveals common DNA methylation patterns of alcohol-associated cancers: A pan-cancer analysis
Source: Front Genet. 2023 Feb 13;14:1032683. doi: 10.3389/fgene.2023.1032683 (PMC9968750; doi:10.3389/fgene.2023.1032683)
Supplement: Supplementary file 2 [file Table5.DOCX]

| **PDMP** | **CHR** | **gene** | **feature** | **cgi** | **status** | **ESCA** | | **HNSC** | | **LIHC** | | **PAAD** | |
| --- | --- | --- | --- | --- | --- | --- | --- | --- | --- | --- | --- | --- | --- |
|  |  |  |  |  |  | **HR** | **pvalue** | **HR** | **.pvalue** | **HR** | **pvalue** | **HR** | **pvalue** |
| cg03679755 | 10 |  | IGR | island | Hyper-  Methylated | 0.1628 | 0.0051 | 0.4353 | 0.0915 | 1.6348 | 0.5254 | 3.9991 | 0.013 |
| cg04021697 | 1 | WDR8 | TSS1500 | island | Hyper-  Methylated | 1.0611 | 0.9399 | 2.7678 | 0.0493 | 3.0635 | 0.0674 | 5.5625 | 0.004 |
| cg07533148 | 1 | TRIM58 | 1stExon | island | Hyper-  Methylated | 0.2034 | 0.0226 | 0.4097 | 0.0296 | 1.6245 | 0.4918 | 4.0981 | 0.0555 |
| cg13356896 | 2 | BOLL | TSS200 | island | Hyper-  Methylated | 0.1765 | 0.0502 | 1.2151 | 0.7227 | 3.607 | 0.0209 | 3.8435 | 0.009 |
| cg15811515 | 16 | CSDAP1 | TSS200 | island | Hyper-  Methylated | 0.1947 | 0.0198 | 0.9314 | 0.937 | 2.522 | 0.0979 | 4.8638 | 0.0243 |
| cg16269733 | 1 | BCAN | Body | island | Hyper-  Methylated | 0.153 | 0.0174 | 0.6129 | 0.3553 | 3.5072 | 0.0168 | 3.8401 | 0.0304 |
| cg16823083 | 1 | WDR8 | TSS1500 | island | Hyper-  Methylated | 0.9579 | 0.9681 | 3.3198 | 0.0924 | 4.0903 | 0.0797 | 5.803 | 0.0267 |
| cg17941572 | 1 |  | IGR | island | Hyper-  Methylated | 0.0696 | 0.0119 | 0.4614 | 0.2373 | 10.2972 | 0.0059 | 3.5668 | 0.0622 |
| cg19497031 | 13 | POU4F1 | Body | island | Hyper-  Methylated | 0.2874 | 0.0482 | 2.0756 | 0.0566 | 1.9952 | 0.3184 | 4.1654 | 0.0283 |
| cg23727983 | 11 | DDX25 | TSS200 | shore | Hyper-  Methylated | 0.2483 | 0.0212 | 2.338 | 0.0288 | 2.956 | 0.0781 | 4.1142 | 0.0164 |
| cg24613080 | 17 | ACCN1 | TSS1500 | island | Hyper-  Methylated | 0.1769 | 0.043 | 0.8419 | 0.8286 | 3.8964 | 0.0798 | 5.9995 | 0.0576 |

**Table S5** The characteristic of eleven survival related PDMPs and HR value in each cancer.
