# Supplementary material for: Integrated analysis reveals common DNA methylation patterns of alcohol-associated cancers: A pan-cancer analysis
Source: Front Genet. 2023 Feb 13;14:1032683. doi: 10.3389/fgene.2023.1032683 (PMC9968750; doi:10.3389/fgene.2023.1032683)
Supplement: Supplementary file 3 [file Table3.DOCX]

**Table S3** The characteristic of 37 PDMP-gene pairs

| **PDMP** | **Gene** | **Methylation** | **Feature** | **Cgi** | **Status** | **ESCA** | | **HNSC** | | **LIHC** | | **PAAD** | |
| --- | --- | --- | --- | --- | --- | --- | --- | --- | --- | --- | --- | --- | --- |
|  |  |  |  |  |  | **PCC** | **pvalue** | **PCC** | **pvalue** | **PCC** | **pvalue** | **PCC** | **pvalue** |
| cg01581084 | OSR2 | Hyper- | Body | shore | HeB | 0.3166 | 9.00E-04 | 0.2298 | <0.0001 | 0.6081 | <0.0001 | -0.0814 | 0.4184 |
| cg06215569 | ALX3 | Hyper- | Body | island | HeB | 0.2568 | 0.0079 | 0.2862 | <0.0001 | 0.411 | <0.0001 | 0.0092 | 0.9272 |
| cg06445348 | ILDR2 | Hyper- | Body | island | HeB | 0.256 | 0.0081 | -0.223 | <0.0001 | 0.429 | <0.0001 | -0.1244 | 0.215 |
| cg09799983 | CYP1B1 | Hyper- | Body | island | HeB | -0.3724 | <0.0001 | -0.2224 | <0.0001 | -0.0988 | 0.2891 | -0.2492 | 0.012 |
| cg11294513 | ZNF154 | Hyper- | Body | island | HeB | -0.2469 | 0.0107 | -0.3542 | <0.0001 | -0.3241 | 4.00E-04 | -0.6221 | <0.0001 |
| cg14754787 | LHX1 | Hyper- | Body | island | HeB | 0.1227 | 0.2102 | 0.2785 | <0.0001 | 0.2104 | 0.0228 | 0.3843 | <0.0001 |
| cg14861089 | TLX1 | Hyper- | Body | island | HeB | 0.334 | 5.00E-04 | -0.0932 | 0.0921 | 0.4568 | <0.0001 | 0.4554 | <0.0001 |
| cg16269733 | BCAN | Hyper- | Body | island | HeB | 0.4394 | <0.0001 | 0.1846 | 8.00E-04 | 0.3878 | <0.0001 | 0.6062 | <0.0001 |
| cg19497031 | POU4F1 | Hyper- | Body | island | HeB | -0.3643 | 1.00E-04 | -0.33 | <0.0001 | -0.1292 | 0.1651 | -0.2441 | 0.0139 |
| cg19760241 | LHX1 | Hyper- | Body | island | HeB | 0.0952 | 0.3316 | 0.2096 | 1.00E-04 | 0.2224 | 0.016 | 0.3483 | 4.00E-04 |
| cg21185289 | TLX2 | Hyper- | Body | island | HeB | 0.2862 | 0.0029 | -0.3381 | <0.0001 | 0.1953 | 0.0349 | 0.2426 | 0.0145 |
| cg25266629 | TLX1 | Hyper- | Body | island | HeB | 0.3207 | 8.00E-04 | -0.1011 | 0.0673 | 0.4368 | <0.0001 | 0.4656 | <0.0001 |
| cg01227537 | ZIC1 | Hyper- | Promotor | island | HeP | -0.2256 | 0.02 | -0.2765 | <0.0001 | 0.3517 | 1.00E-04 | -0.1487 | 0.1378 |
| cg01268824 | ZNF154 | Hyper- | Promotor | shore | HeP | -0.1249 | 0.2021 | -0.3173 | <0.0001 | -0.2787 | 0.0023 | -0.66 | <0.0001 |
| cg01381846 | HOXA9 | Hyper- | Promotor | island | HeP | -0.4702 | <0.0001 | -0.3949 | <0.0001 | 0.2463 | 0.0074 | 0.1587 | 0.1128 |
| cg02401454 | HBQ1 | Hyper- | Promotor | island | HeP | -0.2586 | 0.0074 | -0.4297 | <0.0001 | -0.2403 | 0.0091 | -0.113 | 0.2605 |
| cg05099508 | SPAG6 | Hyper- | Promotor | island | HeP | -0.2183 | 0.0246 | -0.2514 | <0.0001 | -0.1519 | 0.1021 | -0.5312 | <0.0001 |
| cg05661282 | ZNF154 | Hyper- | Promotor | island | HeP | -0.2455 | 0.0112 | -0.3082 | <0.0001 | -0.2229 | 0.0157 | -0.6367 | <0.0001 |
| cg07533148 | TRIM58 | Hyper- | Promotor | island | HeP | -0.5944 | <0.0001 | -0.679 | <0.0001 | -0.1912 | 0.039 | -0.5181 | <0.0001 |
| cg08315202 | NPTX2 | Hyper- | Promotor | island | HeP | -0.2184 | 0.0245 | -0.3857 | <0.0001 | -0.0179 | 0.8483 | -0.4482 | <0.0001 |
| cg08668790 | ZNF154 | Hyper- | Promotor | shore | HeP | -0.1898 | 0.0513 | -0.3247 | <0.0001 | -0.2956 | 0.0012 | -0.6374 | <0.0001 |
| cg13879483 | USP44 | Hyper- | Promotor | island | HeP | -0.4638 | <0.0001 | -0.598 | <0.0001 | -0.3664 | <0.0001 | -0.5672 | <0.0001 |
| cg14587524 | ZNF781 | Hyper- | Promotor | island | HeP | -0.1926 | 0.048 | -0.3204 | <0.0001 | -0.5044 | <0.0001 | -0.6428 | <0.0001 |
| cg15564098 | EVX1 | Hyper- | Promotor | shore | HeP | 0.451 | <0.0001 | 0.2447 | <0.0001 | 0.2858 | 0.0018 | 0.3539 | 3.00E-04 |
| cg17380661 | SIM1 | Hyper- | Promotor | island | HeP | -0.3354 | 4.00E-04 | -0.3436 | <0.0001 | 0.2182 | 0.0181 | -0.3356 | 6.00E-04 |
| cg18279094 | FOXD3 | Hyper- | Promotor | island | HeP | -0.3478 | 3.00E-04 | -0.3323 | <0.0001 | -0.2206 | 0.0168 | -0.3397 | 5.00E-04 |
| cg18932798 | INA | Hyper- | Promotor | island | HeP | -0.6878 | <0.0001 | -0.6854 | <0.0001 | -0.1343 | 0.1489 | -0.6236 | <0.0001 |
| cg18952796 | NPTX2 | Hyper- | Promotor | island | HeP | -0.3287 | 6.00E-04 | -0.3937 | <0.0001 | -0.3401 | 2.00E-04 | -0.4651 | <0.0001 |
| cg19980771 | SLC22A16 | Hyper- | Promotor | island | HeP | -0.3466 | 3.00E-04 | -0.252 | <0.0001 | -0.1578 | 0.0893 | -0.4347 | <0.0001 |
| cg21790626 | ZNF154 | Hyper- | Promotor | island | HeP | -0.2229 | 0.0216 | -0.3087 | <0.0001 | -0.2319 | 0.0119 | -0.7045 | <0.0001 |
| cg23727983 | DDX25 | Hyper- | Promotor | shore | HeP | -0.2121 | 0.0291 | -0.5147 | <0.0001 | -0.1429 | 0.1243 | -0.5525 | <0.0001 |
| cg24368848 | ZSCAN1 | Hyper- | Promotor | island | HeP | -0.2405 | 0.013 | -0.2756 | <0.0001 | -0.2004 | 0.0303 | -0.527 | <0.0001 |
| cg24848035 | RGS22 | Hyper- | Promotor | island | HeP | -0.3737 | <0.0001 | -0.1409 | 0.0106 | -0.2143 | 0.0204 | -0.4938 | <0.0001 |
| cg27049766 | ZNF154 | Hyper- | Promotor | island | HeP | -0.251 | 0.0094 | -0.3277 | <0.0001 | -0.2552 | 0.0055 | -0.6551 | <0.0001 |
| cg27252696 | SIM1 | Hyper- | Promotor | island | HeP | -0.3583 | 2.00E-04 | -0.3475 | <0.0001 | 0.1713 | 0.0648 | -0.3484 | 4.00E-04 |
| cg06968859 | CTNNA2 | Hypo- | Body | opensea | HoB | 0.2937 | 0.0022 | 0.1929 | 4.00E-04 | 0.484 | <0.0001 | 0.3565 | 3.00E-04 |
| cg18291443 | VEPH1 | Hypo- | Body | opensea | HoB | 0.5196 | <0.0001 | 0.059 | 0.2868 | 0.661 | <0.0001 | 0.4599 | <0.0001 |
