# Supplementary material for: Integrated analysis reveals common DNA methylation patterns of alcohol-associated cancers: A pan-cancer analysis
Source: Front Genet. 2023 Feb 13;14:1032683. doi: 10.3389/fgene.2023.1032683 (PMC9968750; doi:10.3389/fgene.2023.1032683)
Supplement: Supplementary file 5 [file Table4.DOCX]

| Motif Theme | Motif ID | Consensus | | p.value | adj.p.value | E.value | Set |
| --- | --- | --- | --- | --- | --- | --- | --- |
| 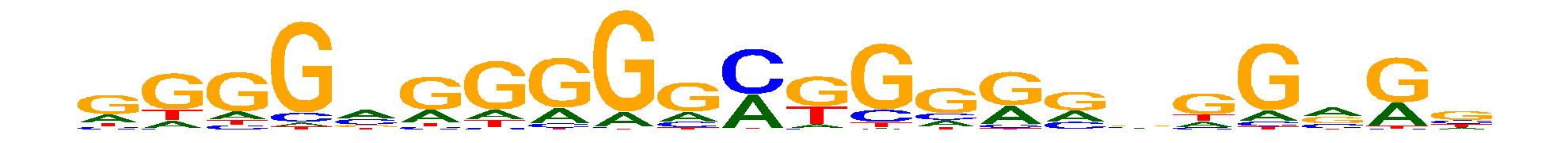 | PATZ1_HUMAN.H11MO.0.C | | GGGGMGGGGGMKGGRRVGGVRG | 1.49E-22 | 8.39E-20 | 3.36E-17 | Hyper- |
| 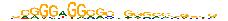 | MAZ_HUMAN.H11MO.0.A | | GGGGGAGGGGGDGRGRRRGRRG | 1.98E-20 | 1.04E-17 | 4.16E-15 | Hyper- |
| 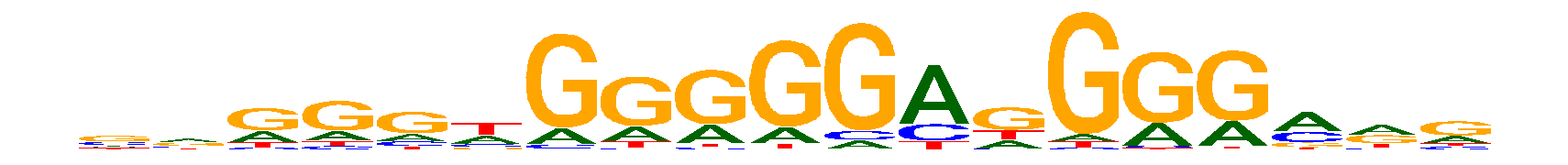 | ZBT17_HUMAN.H11MO.0.A | | SRRGGWGGGGGAGGGGMRR | 9.77E-19 | 5.56E-16 | 2.23E-13 | Hyper- |
| 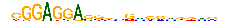 | ZN263_HUMAN.H11MO.0.A | | GGGAGGAGGRRGRGGRGGRR | 3.56E-17 | 1.30E-14 | 5.22E-12 | Hyper- |
| 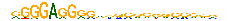 | ZN467_HUMAN.H11MO.0.C | | GGGGAGGGGRRGGRGRRGGRRR | 6.23E-17 | 2.02E-14 | 8.11E-12 | Hyper- |
| 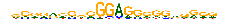 | VEZF1_HUMAN.H11MO.0.C | | GGRRRRGRRGGAGGGGGRGRRR | 8.19E-17 | 4.62E-14 | 1.85E-11 | Hyper- |
| 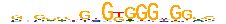 | EGR2_HUMAN.H11MO.0.A | | GRGRRKGWGKGGGHGGRG | 2.18E-16 | 8.20E-14 | 3.29E-11 | Hyper- |
| 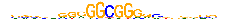 | SP2_HUMAN.H11MO.0.A | | GGSSVGGGGGCGGGGCCDGSGS | 1.62E-16 | 8.33E-14 | 3.34E-11 | Hyper- |
| 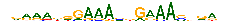 | IRF3_HUMAN.H11MO.0.B | | RAAARGGAAAVDGAAASDGA | 6.04E-16 | 2.01E-13 | 8.06E-11 | Hyper- |
| 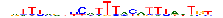 | Z354A_HUMAN.H11MO.0.C | | RTTTARWYCATTTACATTTAATGT | 1.48E-15 | 2.41E-13 | 9.65E-11 | Hyper- |
| 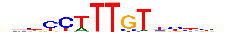 | SOX2_HUMAN.H11MO.0.A | | BBCCTTTGTYYYB | 9.14E-16 | 4.05E-13 | 1.62E-10 | Hyper- |
| 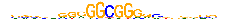 | SP4_HUMAN.H11MO.0.A | | SRGVARGRGGCGGRGCHDRR | 4.37E-15 | 2.33E-12 | 9.34E-10 | Hyper- |
| 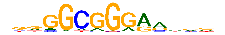 | E2F7_HUMAN.H11MO.0.B | | GDGGCGGGAARDR | 4.36E-15 | 2.40E-12 | 9.63E-10 | Hyper- |
| 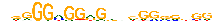 | KLF15_HUMAN.H11MO.0.A | | RGGGMGGRGVDGGGGGRGG | 8.63E-15 | 3.09E-12 | 1.24E-09 | Hyper- |
| 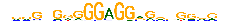 | WT1_HUMAN.H11MO.0.C | | RGGVGGGGGAGGRGGVGGRG | 1.74E-14 | 6.93E-12 | 2.78E-09 | Hyper- |
| 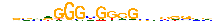 | KLF6_HUMAN.H11MO.0.A | | GSRRGGGHGGGGMHGGGRV | 3.45E-14 | 1.83E-11 | 7.35E-09 | Hyper- |
| 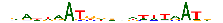 | NKX61_HUMAN.H11MO.0.B | | GAYWAATKRSHWWTTAATK | 1.33E-13 | 3.51E-11 | 1.41E-08 | Hyper- |
| 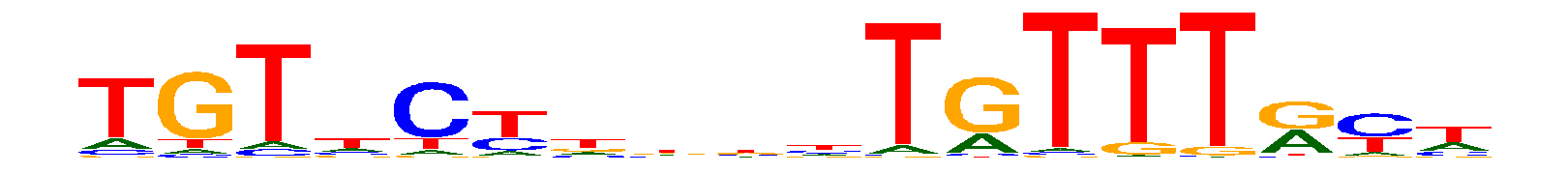 | ANDR_HUMAN.H11MO.0.A | | TGTTCTTKKYTGTTTRYW | 2.08E-13 | 4.91E-11 | 1.97E-08 | Hyper- |
| 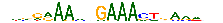 | STAT2_HUMAN.H11MO.0.A | | RRGRAAAHGAAACTGAAAV | 9.00E-13 | 1.55E-10 | 6.21E-08 | Hyper- |
| 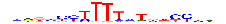 | ZN350_HUMAN.H11MO.0.C | | CAGYYYTTTTATDWCCHR | 3.86E-13 | 1.71E-10 | 6.84E-08 | Hyper- |
| 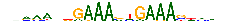 | IRF1_HUMAN.H11MO.0.A | | RAAANWGAAASTGAAASYRR | 1.59E-12 | 2.42E-10 | 9.71E-08 | Hyper- |
| 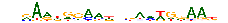 | ZN394_HUMAN.H11MO.0.C | | NRARWRGAAWNGAMWGRAAK | 8.94E-13 | 3.90E-10 | 1.56E-07 | Hyper- |
| 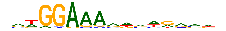 | NFAC1_HUMAN.H11MO.0.B | | AWGGAAARWVWGAMW | 1.52E-12 | 7.10E-10 | 2.85E-07 | Hyper- |
| 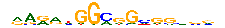 | TAF1_HUMAN.H11MO.0.A | | RARRWGGCGGMGGMGR | 1.66E-12 | 9.06E-10 | 3.63E-07 | Hyper- |
| 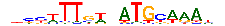 | NANOG_HUMAN.H11MO.0.A | | BYYWTTGWNATGCAAAT | 5.75E-12 | 1.62E-09 | 6.48E-07 | Hyper- |
| 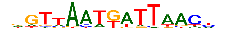 | HNF1A_HUMAN.H11MO.0.C | | DGTTAATKATTAACH | 2.75E-11 | 5.03E-09 | 2.02E-06 | Hyper- |
| 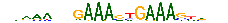 | IRF2_HUMAN.H11MO.0.A | | RAAAVHGAAAGTGAAASTRV | 4.76E-11 | 5.75E-09 | 2.31E-06 | Hyper- |
| 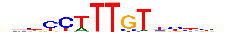 | SOX4_HUMAN.H11MO.0.B | | BYCTTTGTYYYB | 1.63E-11 | 7.35E-09 | 2.95E-06 | Hyper- |
| 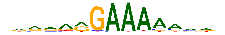 | PRDM6_HUMAN.H11MO.0.C | | RRRARGAAAAAAA | 2.75E-11 | 1.26E-08 | 5.05E-06 | Hyper- |
| 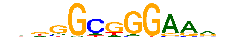 | E2F3_HUMAN.H11MO.0.A | | DDGGCGGGAAA | 2.81E-11 | 1.49E-08 | 5.99E-06 | Hyper- |
| 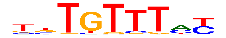 | FOXP1_HUMAN.H11MO.0.A | | TBTGTTTMY | 3.62E-11 | 1.56E-08 | 6.24E-06 | Hyper- |
| 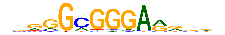 | E2F6_HUMAN.H11MO.0.A | | DGGGCGGGARRVR | 3.92E-11 | 2.07E-08 | 8.29E-06 | Hyper- |
| 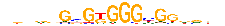 | EGR1_HUMAN.H11MO.0.A | | VDRDGCGKGGGYGGRRR | 5.99E-11 | 2.16E-08 | 8.67E-06 | Hyper- |
| 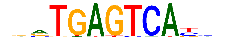 | JUN_HUMAN.H11MO.0.A | | DRTGAGTCAYH | 9.84E-22 | 1.61E-19 | 6.47E-17 | Hypo- |
| 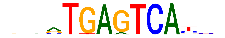 | FOSL2_HUMAN.H11MO.0.A | | NDRTGAGTCAYH | 1.10E-20 | 1.49E-18 | 5.96E-16 | Hypo- |
| 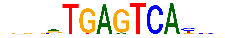 | FOSL1_HUMAN.H11MO.0.A | | KRVTGAGTCAYH | 3.04E-19 | 4.14E-17 | 1.66E-14 | Hypo- |
| 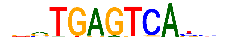 | JUND_HUMAN.H11MO.0.A | | RRTGAGTCAYY | 3.04E-19 | 6.18E-17 | 2.48E-14 | Hypo- |
| 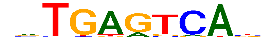 | FOSB_HUMAN.H11MO.0.A | | RTGAGTCAB | 9.73E-19 | 2.63E-16 | 1.05E-13 | Hypo- |
| 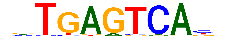 | FOS_HUMAN.H11MO.0.A | | VTGAGTCAB | 2.55E-17 | 6.29E-15 | 2.52E-12 | Hypo- |
| 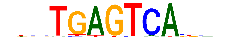 | JUNB_HUMAN.H11MO.0.A | | DVTGAGTCABH | 2.78E-15 | 5.95E-13 | 2.39E-10 | Hypo- |

**Table S4** The characteristics of 33 hyper- and 7 hypomethylated TF motifs
