# Supplementary material for: Integrated analysis reveals common DNA methylation patterns of alcohol-associated cancers: A pan-cancer analysis
Source: Front Genet. 2023 Feb 13;14:1032683. doi: 10.3389/fgene.2023.1032683 (PMC9968750; doi:10.3389/fgene.2023.1032683)
Supplement: Supplementary file 6 [file DataSheet1.docx]

**
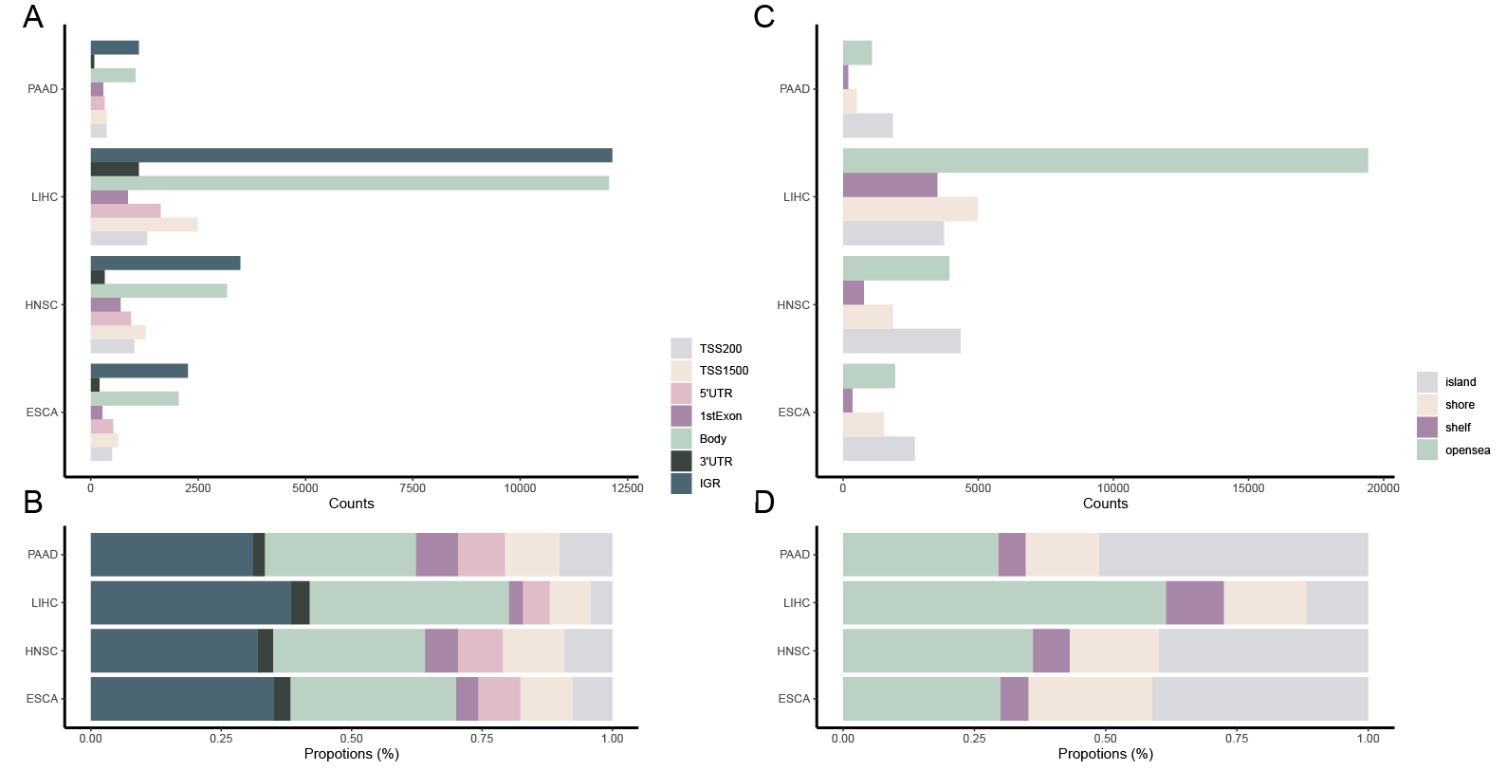
**

**Supplementary Figure S1** The characteristics of differentially methylated probes (DMPs). **A-B.** The distribution of DMPs in featured regions. **C-D.** The distribution of DMPs in CpG regions.

**
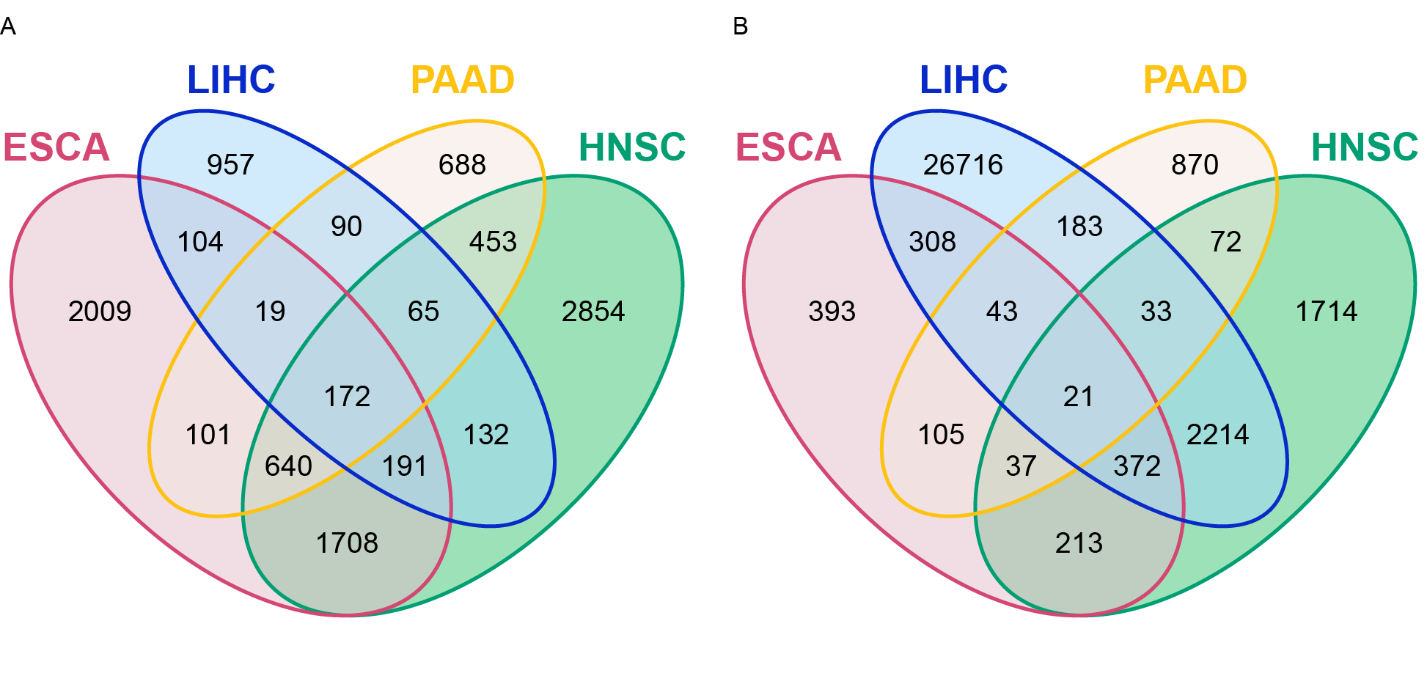
**

**Supplementary Figure S2** The venn plot showed the hyper- and hypomethylated PDMPs in 4 alcohol-associate cancers.

**
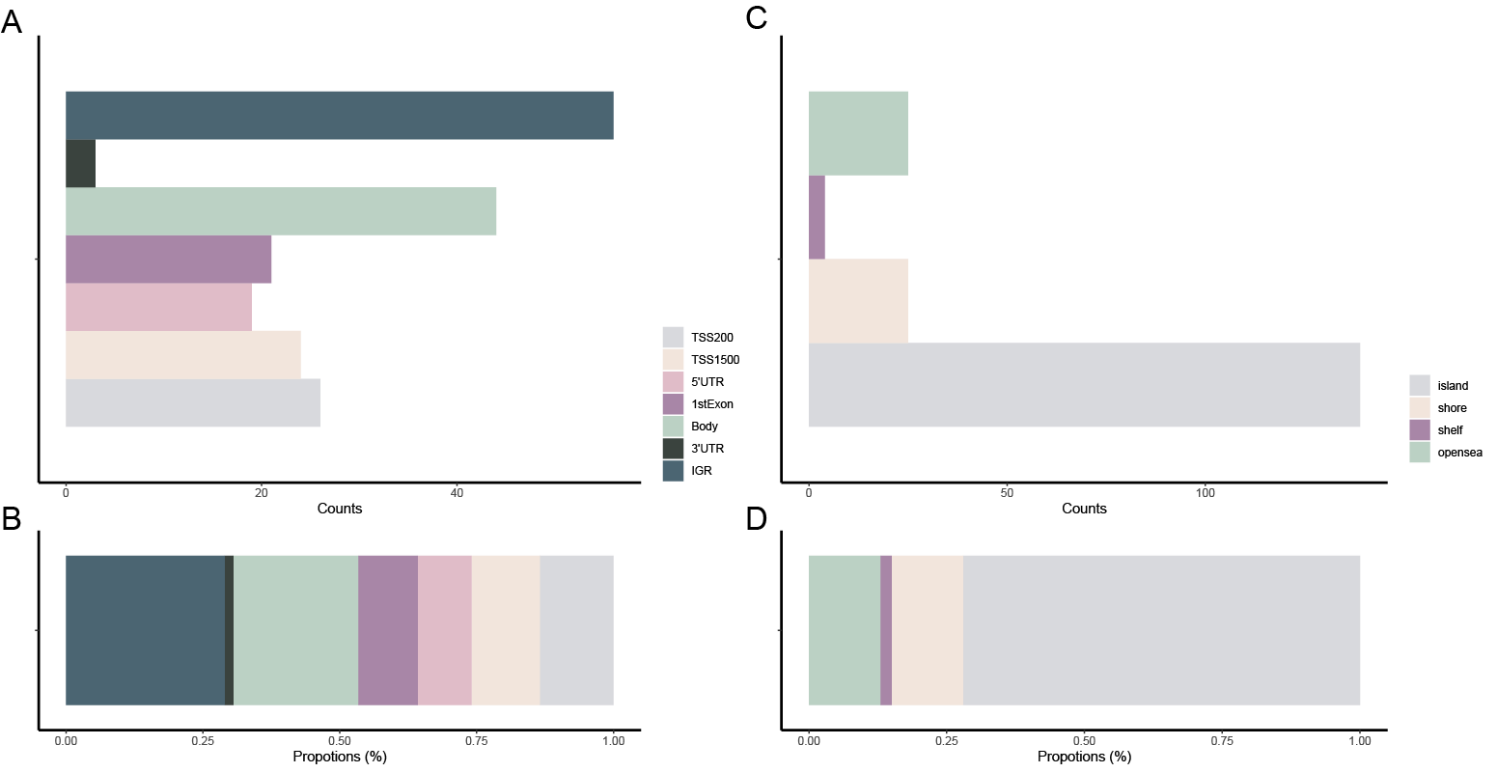
**

**Supplementary Figure S3** The distribution of PDMPs in featured (**A and B**) and CpG regions (**C and D**).

**
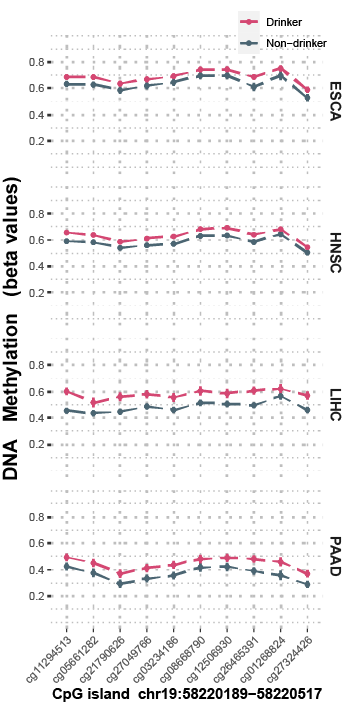
**

**Supplementary Figure S4** The beta values of CpG probes in CpG island: *chr19:58220189-58220517* in 4 alcohol-associated cancers.

**
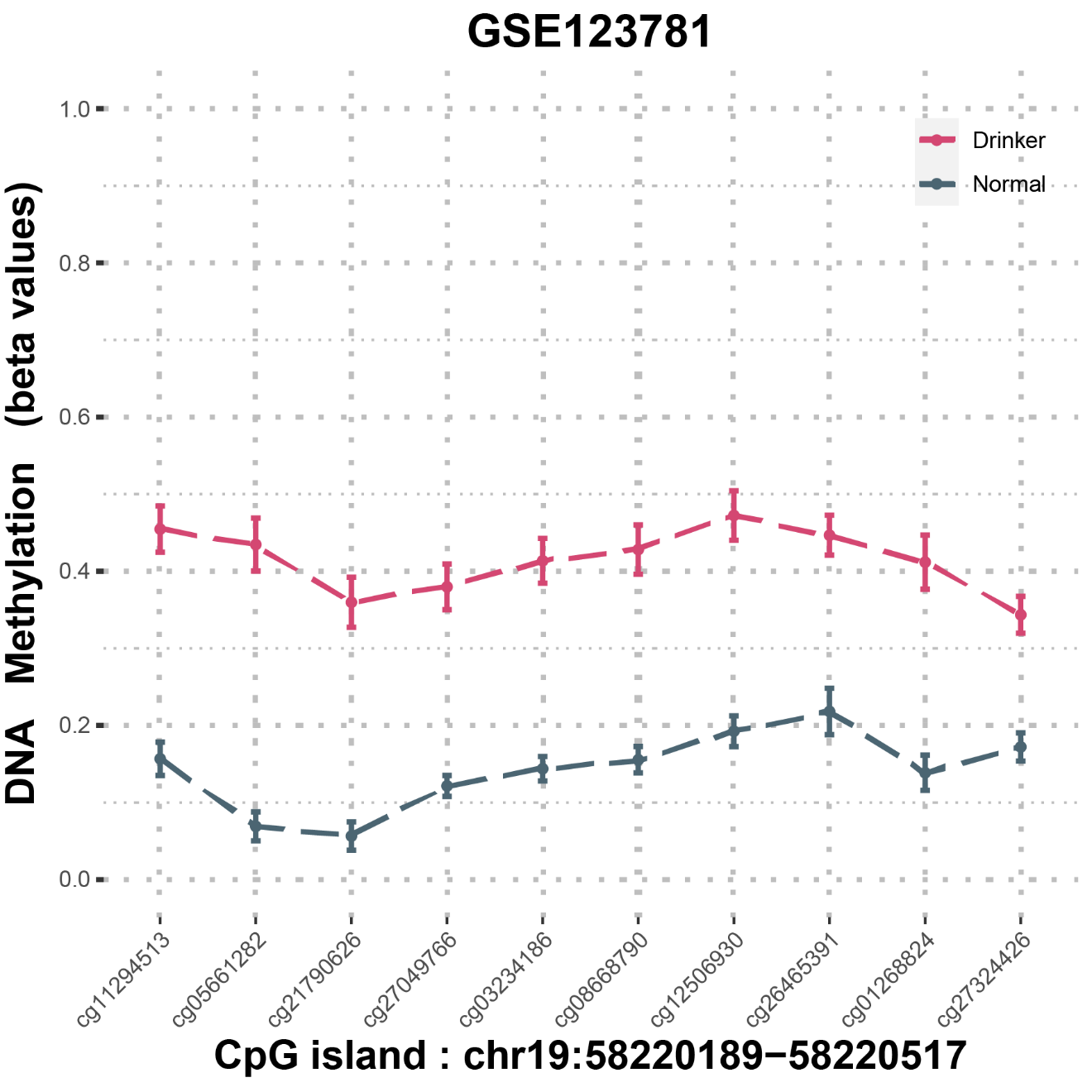
**

**Supplementary Figure S5** The beta values of CpG probes in CpG island: *chr19:58220189-58220517* in GSE123781.

**
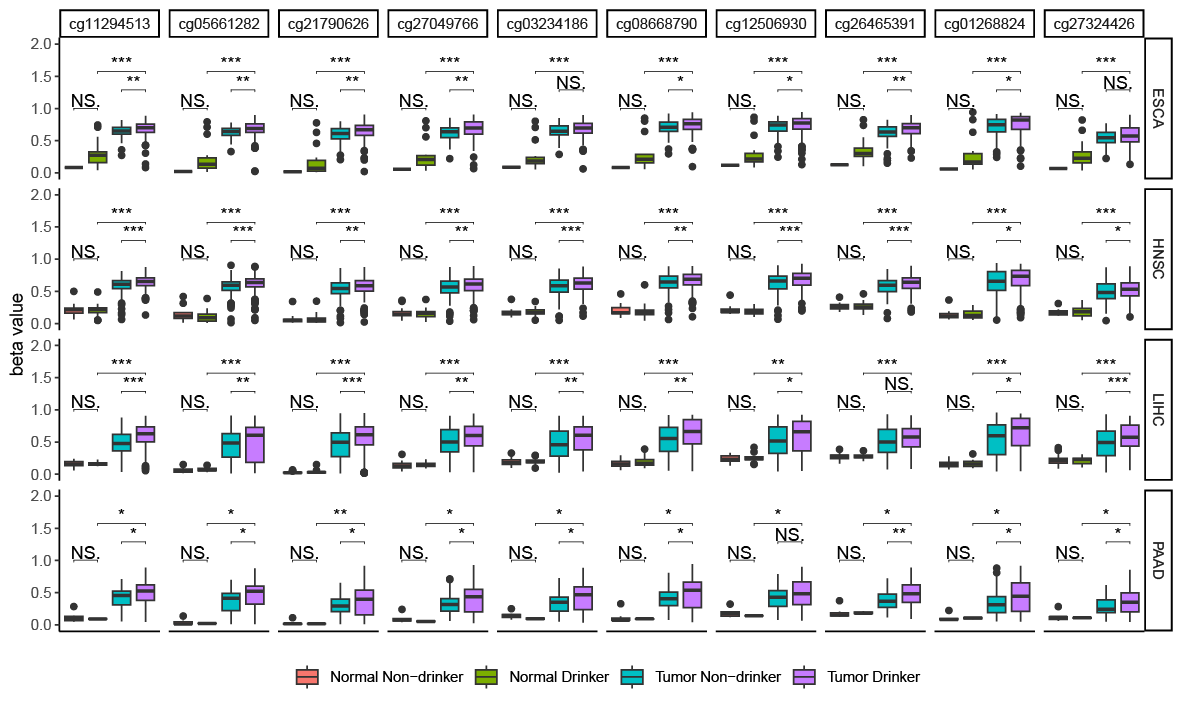
**

**Supplementary Figure S6** The beta values of CpG probes in CpG island: *chr19:58220189-58220517* were divided into 4 groups based on tissue origin and alcohol consumption.

**
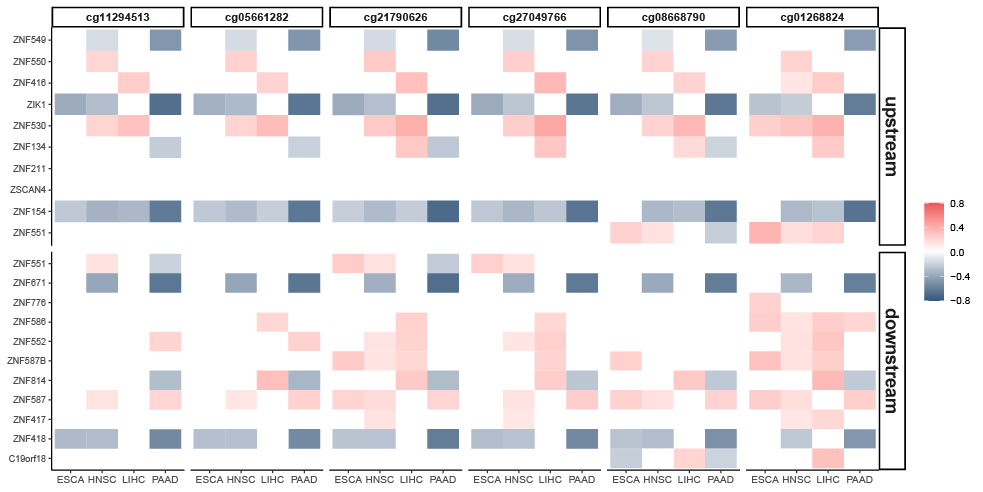
**

**Supplementary Figure S7** The Pearson Coefficient Correlations (PCCs) were estimated between PDMPs and the closet 10 upstream/downstream genes separately.
